# Biologic Augmentation With Biocomposite Scaffold in Revision Quadriceps Tendon Repair

**DOI:** 10.1002/atn2.70021

**Published:** 2026-06-06

**Authors:** Cory N. Meixner, Emma L. Flanigan, David C. Flanigan

**Affiliations:** ^1^ Orthopaedic Surgery and Sports Medicine The Ohio State University Wexner Medical Center Columbus Ohio U.S.A.; ^2^ Sports Medicine Research Institute The Ohio State University Wexner Medical Center Columbus Ohio U.S.A.

## Abstract

Quadriceps tendon rupture is a relatively uncommon injury, typically occurring in middle‐aged males, and is often associated with systemic comorbidities or trauma. Although primary repair techniques provide excellent outcomes when performed acutely, revision surgery presents unique challenges due to poor tissue quality and compromised vascularity. This article outlines a step‐by‐step technique for revision quadriceps tendon repair incorporating transosseous fixation, suture cerclage, and biologic augmentation with BioBrace.

## Graphical abstract

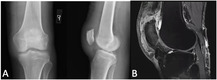

**VIDEO 1:** Revision quadriceps tendon repair with biological augmentation with BioBrace. Left knee. The patient is positioned supine with a lateral padded thigh post and Kirschenbaum foot positioned. After transosseous quadriceps repair and circumferential suture cerclage, biologic augmentation is performed with a ConMed BioBrace (ConMed, Largo, FL) shoelace soaked in vancomycin with shuttling sutures at both ends; and a ConMed BioBrace patch soaked in platelet‐rich plasma. First, the shoelace creates a circumferential cerclage, weaving proximal to the previously placed Krakow sutures, under the quadriceps tendon, and along the medial and lateral margins of the patella. Both ends are retrieved underneath the patellar tendon, tensioned with the knee in hyperextension, and secured with a combination of FiberWire and Vicryl figure‐of‐eight sutures. The platelet‐rich plasma‐soaked patch is then laid over the repair construct for further biologic augmentation. It is first secured with simple Vicryl sutures at the corners, then with evenly spaced simple sutures along all sides to help the patch lie flat without any bunching. Video content can be viewed at https://doi.org/10.1002/atn2.70021.

Quadriceps tendon ruptures are rare, with an incidence of 1.37 per 100,000 annually,^1^ with primary injury often due to indirect trauma or systemic comorbidities. Although acute repair using transosseous tunnels or suture anchors yields excellent outcomes, rerupture rates are between 2% and 8%, and revision surgery after failed repair remains a challenge due to retraction, scarring, and tissue degeneration.^1^, ^2^, ^3^

Recent literature supports the use of biologic augmentation to enhance healing in compromised tissue beds.^4^, ^5^, ^6^, ^7^ However, long‐term and meaningful outcomes are limited. This article describes a revision quadriceps tendon repair technique that combines robust mechanical fixation strategies—transosseous repair with Arthrex #2 FiberWire (Arthrex, Naples, FL) facilitated by Mitek 2.4‐mm Beath pins (DePuy Mitek, Raynham, MA) and circumferential suture cerclage with Arthrex #5 FiberWire—with biologic augmentation using ConMed BioBrace (ConMed, Largo, FL), designed to enhance healing and reduce the risk of recurrent rerupture in compromised tissue environments.

## SURGICAL TECHNIQUE

### Patient Evaluation

**FIGURE 1:**
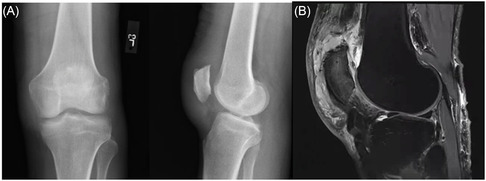
Left knee imaging 11 days after suture anchor quadriceps tendon repair following a fall. (A) A‐P and lateral radiographs show an effusion and recurrent patella baja with a Caton‐Deschamps index of 0.65. (B) Representative T2‐weighted sagittal MRI cut showing a complete retear of the distal quadriceps tendon. (MRI, magnetic resonance imaging; A‐P, anterior‐posterior.)

A detailed patient evaluation should be completed for patients with new‐onset extensor mechanism deficit after previous quadriceps tendon repair. This should begin with a detailed history and physical examination. A detailed history should address several key factors: the mechanism of injury, the time since the index surgery/reinjury, and the patient's activity level/function. It should also include medical comorbidities and smoking status. On physical examination, patients often present with an extensor lag, inability to perform a straight leg raise, and/or a palpable defect. If it is an acute reinjury, an effusion may also be present. Radiographs should be obtained and carefully evaluated for patellar height (i.e., baja) and, if ongoing concern, confirmed with an magnetic resonance imaging to assess tendon discontinuity, tissue quality, and potential reducibility (Figure 1).

### Indications

Revision surgery is indicated for failed index quadriceps tendon repair with extensor mechanism deficit including patella baja, extensor lag, and/or palpable quadriceps defect. Magnetic resonance imaging is not vital but can confirm the diagnosis and help approximate tissue reducibility and the applicability of this biologic augmentation technique. Biologic augmentation is indicated for poor tissue quality and reduced vascularity, especially in the context of patient comorbidities—chronic renal failure, rheumatoid arthritis, diabetes, gout, a history of steroid use^1^—or chronicity >3 weeks.^1^, ^3^

### Surgical Technique

The patient is placed in the supine position with a bump under the ipsilateral hip to facilitate neutral leg rotation with the patella facing directly upward. A tourniquet is applied but not routinely inflated. A lateral thigh post and Kirschenbaum foot positioner or sandbag is placed to allow the leg to rest unassisted at approximately 45° of knee flexion.

**FIGURE 2:**
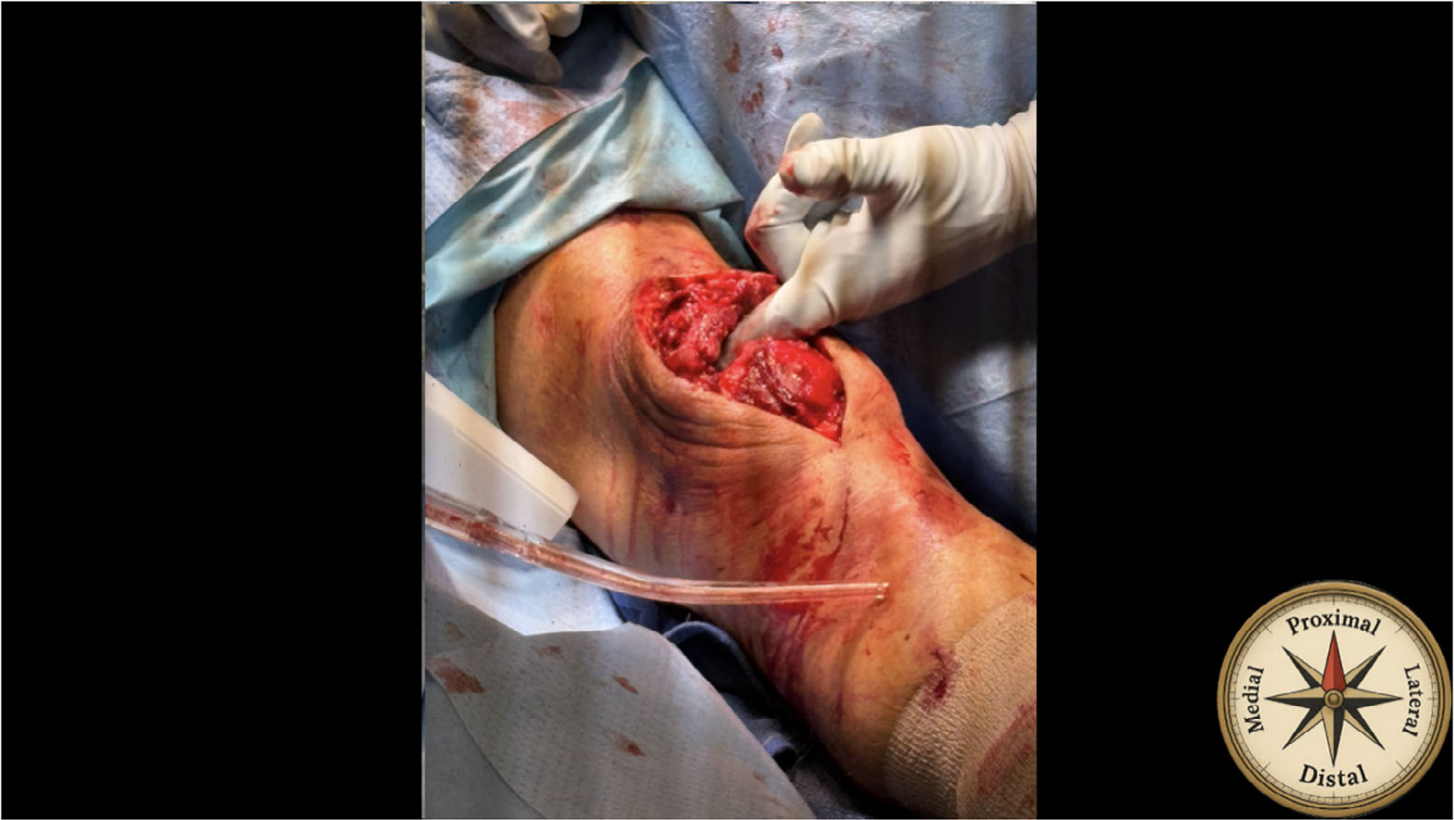
Intraoperative image of a supine patient's left knee. After foreign bodies from the index surgery as well as scar and devitalized tissue are removed, the quadriceps tendon retear is thoroughly evaluated for tendon quality and reticular tears.

The midline incision is recreated, ensuring it extends roughly 3 fingerbreadths proximal to the superior pole of the patella to the tibial tubercle (Video 1). Full‐thickness soft‐tissue flaps are raised medially and laterally. Hematoma is evacuated to allow identification of the tear site. All foreign bodies from the previous repair are removed, as is scar and devitalized tissue. The wound is copiously irrigated, and the tear is thoroughly evaluated for tendon quality and retinacular tears (Figure 2).

Two Arthrex #2 FiberWire sutures are passed up the medial and lateral margins of the quadriceps tendon, respectively, for 4 to 5 throws in a modified locking Krackow fashion and then back down centrally. The tear is assessed for candidacy of biologic augmentation.

Once deemed necessary, a ConMed BioBrace shoelace (5 × 250 mm) is prepared by placing 0 Vicryl sutures at both ends of the shoelace in a modified Krackow fashion to serve as shuttling sutures. It is then soaked in vancomycin and a ConMed BioBrace patch (40 × 60 mm) in platelet‐rich plasma on the back table.

**FIGURE 3:**
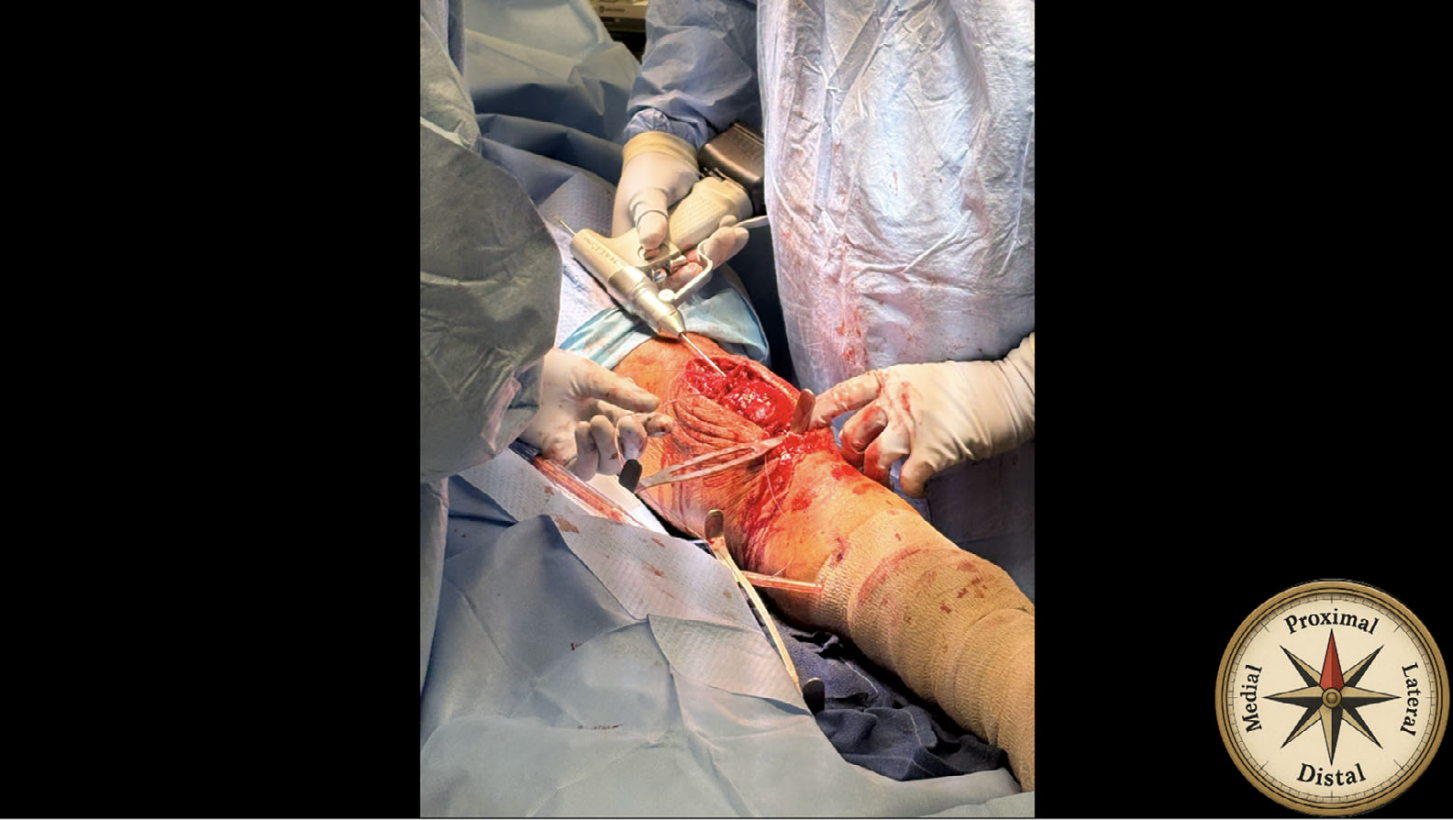
Intraoperative image of a supine patient's left knee showing a Beath pin creating the central transosseous tunnel, which then serves as a guide for the medial and lateral Beath pins. These pins then shuttle previously placed FiberWire Krakow suture tails within the quadriceps tendon with 1 medial, 1 lateral, and 2 central. The central suture tails are retrieved beneath the patellar tendon to their respective medial and lateral complements. With the knee in hyperextension, they are tensioned and then tied over the inferior pole of the patella.

With the knee flexed 45°, 2 Mitek 2.4‐mm Beath pins facilitate creation of 3 parallel tunnels 1 cm apart in an anterograde fashion through the center, medial, and lateral aspects of the patella. The central pin is placed first and left in place, serving as a guide for the medial and lateral pins. These pins subsequently shuttle the previously placed Arthrex #2 FiberWire suture tails distally with 1 medial, 1 lateral, and 2 central. The central Arthrex #2 FiberWire suture tails are retrieved beneath the patellar tendon to their respective medial and lateral complements (Figure 3).

A circumferential suture cerclage is created with Arthrex #5 FiberWire around the patella using the “Wisconsin technique.”^8^ The Arthrex #5 FiberWire suture cerclage should be extended proximal to the previously placed quadriceps Krackow sutures when traversing the quadriceps tendon.

**FIGURE 4:**
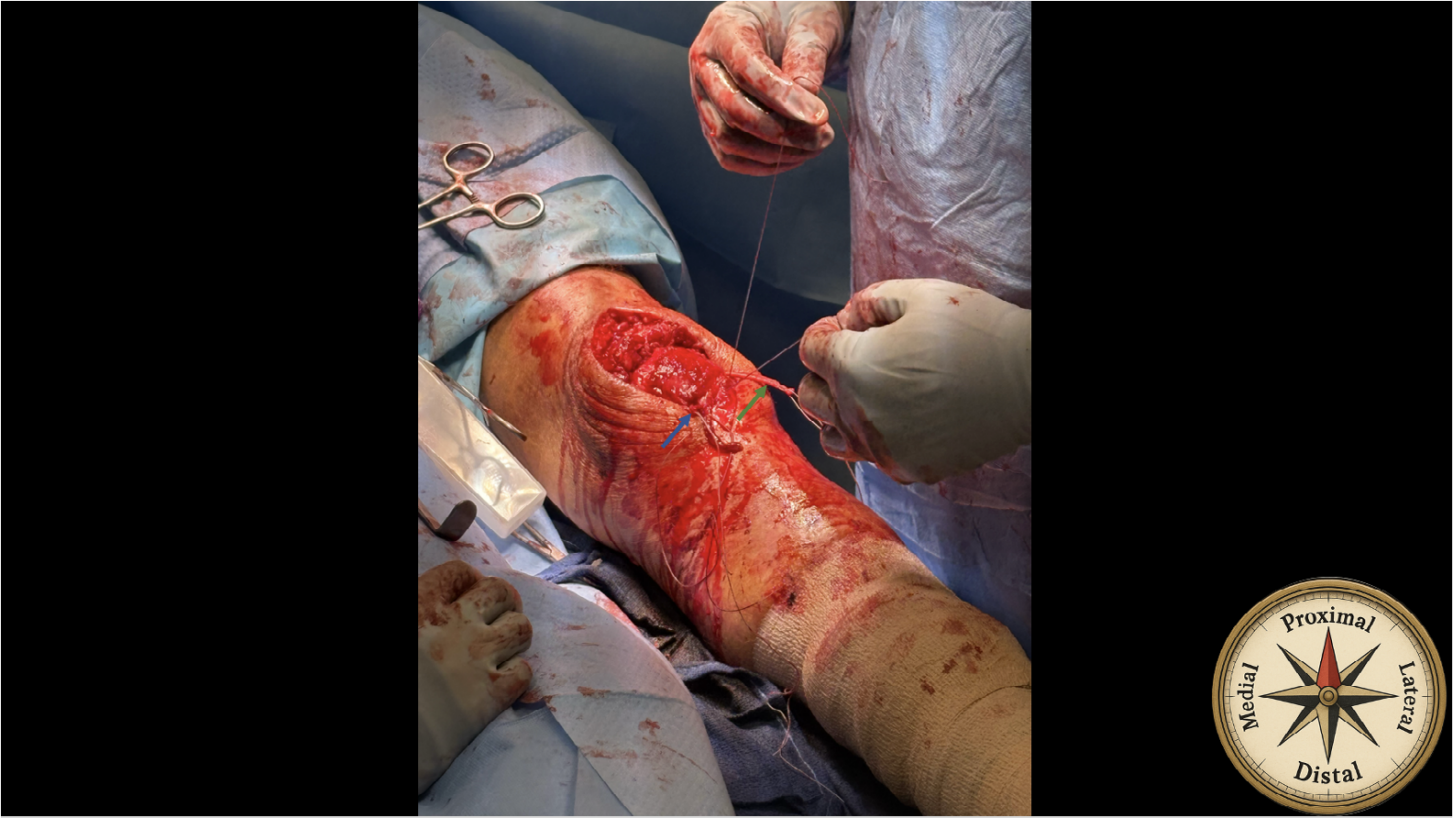
Intraoperative image of a supine patient's left knee showing a vancomycin‐soaked ConMed BioBrace shoelace, which has been weaved proximally, proximal to the previously placed Krakow sutures and beneath the quadriceps tendon. The medial (blue arrow) and lateral (green arrow) limbs of the shoelace are weaved distally adjacent to the patella and patellar tendon. Both ends are retrieved underneath the patellar tendon with a right‐angle clamp. With the knee in hyperextension, they are tensioned and then secured with FiberWire figure‐of‐eight sutures and reinforced with a series of Vicryl figure‐of‐eight sutures.

The ConMed BioBrace shoelace is weaved around the patella, starting proximally, beneath the quadriceps tendon but proximal to the previously placed quadriceps Krackow sutures. This is facilitated by a right‐angle clamp and the passing suture limbs on the ends of the shoelace. This is then weaved distally on the medial and lateral margins adjacent to the tendon and patella, again using a right‐angle clamp (Figure 4).

The quadriceps tendon is reduced by placing the knee in hyperextension and applying tension to the ConMed BioBrace shoelace. The transosseous Arthrex #2 FiberWire sutures are tied over the inferior pole of the patella. The Arthrex #5 FiberWire suture cerclage is tied on the lateral aspect.

The medial and lateral retinacular tears are repaired with 0 Vicryl figure‐of‐eight sutures. To easily access the far medial and lateral aspects of the torn retinaculum, it can be helpful to place those sutures from the opposite side of the patient (i.e., medial sutures placed from the patient's lateral side and vice versa).

Both ends of the previously weaved ConMed BioBrace shoelace are retrieved underneath the patellar tendon with a right‐angle clamp. With the knee maintained in hyperextension to keep the tension off the repair construct, the shoelace is tensioned and then secured with FiberWire figure‐of‐eight sutures on the medial and lateral sides of the patella and reinforced with a series of Vicryl figure‐of‐eight sutures.

**FIGURE 5:**
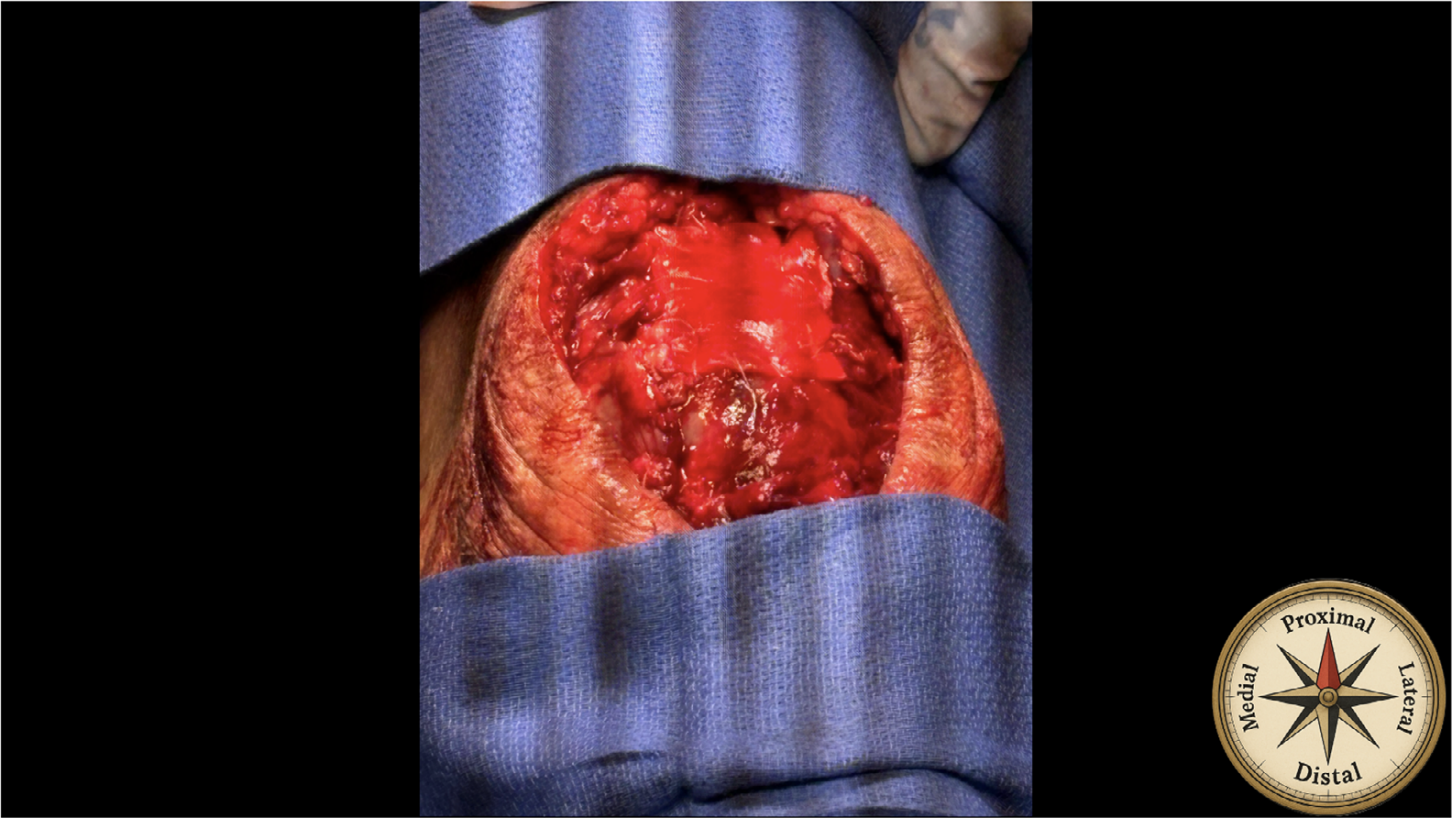
Intraoperative image of a supine patient's left knee showing a platelet‐rich plasma–soaked ConMed BioBrace patch laid over the quadriceps repair construct and secured with simple 0 Vicryl sutures. The final repair construct is composed of transosseous quadriceps repair, circumferential suture cerclage, and biologic augmentation with a circumferential BioBrace solace cerclage and overlying BioBrace patch.

The ConMed BioBrace patch is laid over the quadriceps repair construct and secured with simple 0 Vicryl sutures—first at the corners and then evenly spaced around the patch to minimize bunching (Figure 5).

**FIGURE 6:**
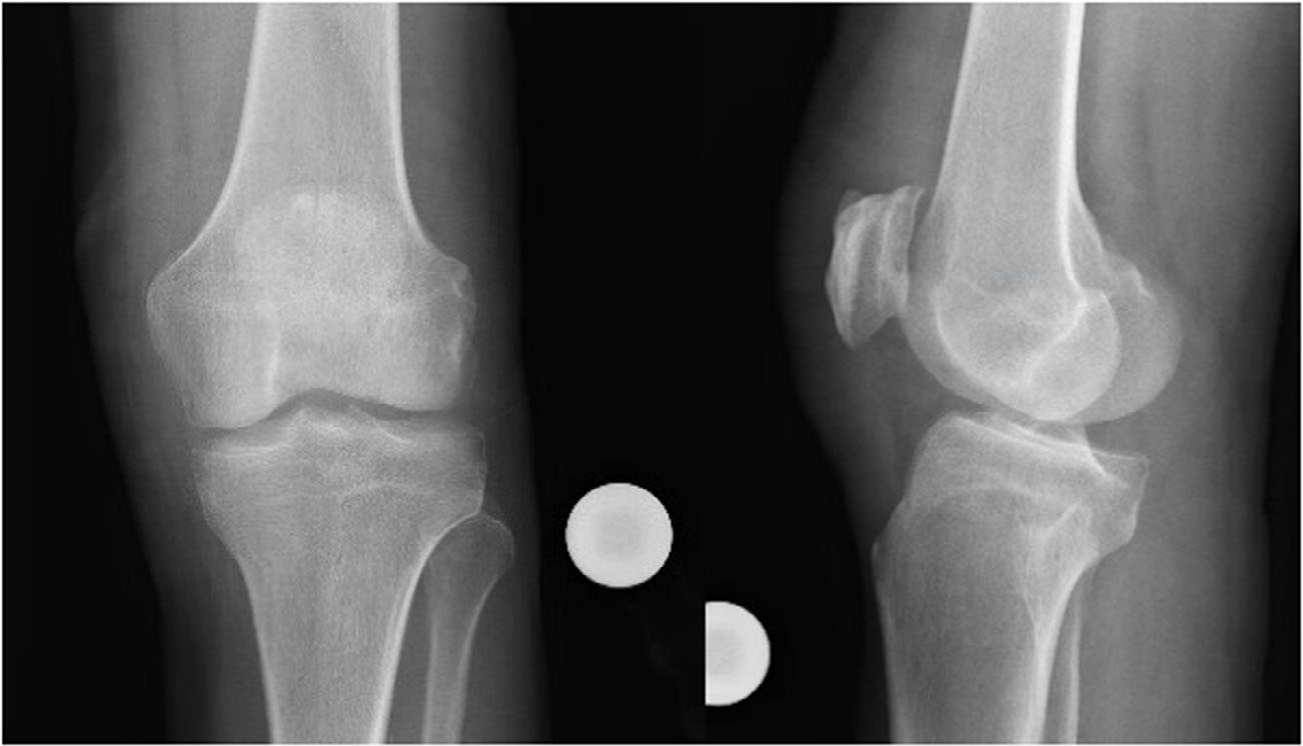
Left knee A‐P and lateral radiographs status postrevision quadriceps tendon repair with biologic augmentation show normalization of patellar heights with a Caton‐Deschamps index of 0.91. (A‐P, anterior‐posterior.)

The wound is copiously irrigated with sterile saline. A layered closure is performed with deep 0 Vicryl, buried 2‐0 Vicryl for deep dermal reapproximation, and a running 3‐0 Prolene for subcuticular closure. Sterile dressings are applied in addition to a cooling unit, ACE bandage, and knee immobilizer locked in extension (Figure 6).

### Rehabilitation

Postoperatively, the patient is allowed to weight‐bear as tolerated with a hinged knee brace locked in extension at all times for the first week. Thereafter, they can unlock the knee brace from 0 to 70° while at rest. Patients are prescribed 6 weeks of aspirin for deep‐vein thrombosis prophylaxis. From 6 to 12 weeks, range of motion is incrementally increased in an unlocked hinged knee brace, with the goal of full range of motion by 3 months postoperatively. From 3 months onward, the precedent is placed on strengthening, specifically eccentric quadriceps exercises, followed by progression of activities as tolerated.

## DISCUSSION

Revision quadriceps tendon repairs are technically demanding due to the compromised healing environment—patients often have comorbidities such as chronic renal failure, rheumatoid arthritis, diabetes, gout, or a history of steroid use^1^; quadriceps tendon ruptures most frequently occur at the vascular watershed area 1 to 2 cm proximal to the patella^9^; and revision settings entail further tissue degeneration, scar formation, tendon retraction, and vascular compromise.^4^, ^6^

The method of fixation for index repair is a topic of ongoing debate.^1^, ^2^, ^3^, ^10^ Transosseous tunnels provide strong fixation but require greater surgical exposure, whereas suture anchors allow for less invasive repair and preservation of vascularity but may carry higher costs and rerupture rates.^2^ Although there is no significant difference in functional outcomes between the 2 methods, there has been a higher complication rate with suture anchors (9.3%) compared with transosseous tunnels (1.3%).

In revision settings, conventional repair techniques may not provide sufficient fixation or healing potential. Consequently, augmentation has become a key strategy in managing these difficult cases. Options include hamstring autografts, Achilles allografts, patellar‐quadriceps allografts, Prolene mesh, and biologic materials,^4^, ^5^, ^6^, ^7^ but meaningful outcomes are limited and not generalizable to different techniques.

This technique incorporates biologic augmentation with ConMed BioBrace providing structural and cellular support to the repair, while transosseous fixation and suture cerclage ensure robust mechanical stability. This multimodal approach addresses the limitations of previous repair methods and provides a reproducible technique for challenging revision cases.

Pearls and pitfalls of this technique are presented in Table 1, and advantages and disadvantages are listed in Table 2. A demonstration of this technique on a patient's left knee is shown in Video 1.

**TABLE 1:** Pearls and Pitfalls

Preoperative magnetic resonance imaging can be helpful in approximating tendon reducibility, as this is necessary for this augmentation technique
Careful placement of Beath pins is necessary to maximize fixation strength and tendon tensioning. This may be compromised with superficial or deep cortical penetration or misaligned/converging tunnels Place the central Beath pin first and leave in place to serve as a guide for the medial and lateral tunnels. This can be done freehand referencing preoperative imaging or with intraoperative fluoroscopy
Inadequate length‐tension restoration may lead to an extensor lag Place the knee in hyperextension while tensioning the repair construct If using a tourniquet, consider deflation while tensioning
Minimize wound complications through meticulous hemostasis, watertight layered closure, and use of a hinged knee brace locked in extension for initial soft‐tissue rest Consider longer immobilization/slower progression if tissue or bone quality is especially poor Casts are seldom used but are reserved for truly nonadherent patients or those at an especially high fall risk for added protection

**TABLE 2:** Advantages and Limitations

Advantages	Limitations
Minimal additional invasiveness	Necessitates reducible tendon and adequate bone quantity If irreducible even after release of medial/lateral adhesions, freeing vastus intermedius from anterior femur, and/or formal VY‐lengthening, may favor allograft reconstruction If inadequate bone quantity preventing creation of new bone tunnels, this may also favor allograft reconstruction
Multifaceted mechanical fixation: transosseous repair and circumferential suture cerclage Multifaceted biocomposite augmentation: vancomycin‐soaked biocomposite shoelace cerclage, platelet‐rich plasma–soaked biocomposite patch	Long‐term outcomes are limited
May reduce retear rates	Additional cost of implants
No donor site morbidity No allograft‐related risks (infection, disease transmission, graft vs host disease)	

## DISCLOSURES

The author (D.C.F.) declares the following financial interests/personal relationships, which may be considered as potential competing interests: D.C.F. reports a relationship with CONMED Corporation and Smith & Nephew that includes consulting or advisory; reports a relationship with Vericel that includes consulting or advisory and funding grants; reports a relationship with Anika Therapeutics Inc and DePuy Synthes Mitek Sports Medicine that includes consulting or advisory; reports a relationship with Moximed Inc that includes consulting or advisory, equity or stocks, and funding grants; reports a relationship with Hyalex Orthopaedics that includes equity or stocks; reports a relationship with Arcuro Medical that includes equity or stocks. Associate Editor: *J Knee Surgery*. The other authors (C.N.M., E.L.F.) declare that they have no known competing financial interests or personal relationships that could have appeared to influence the work reported in this article.
